# The association between social integration and utilization of primary health care among migrants in China: a nationwide cross-sectional study

**DOI:** 10.1186/s12939-023-02018-x

**Published:** 2023-10-09

**Authors:** Xueyao Wang, Jue Liu, Jingmin Zhu, Yang Bai, Jun Wang

**Affiliations:** 1 Center for Health Policy Research and Evaluation, Renmin University of China, Beijing, 100872 China; 2 School of Public Administration and Policy, Renmin University of China, Beijing, 100872 China; 3 Department of Epidemiology and Biostatistics, School of Public Health, Peking University Health Science Centre, Beijing, 100080 China; 4 Institute for Global Health and Development, Peking University, Beijing, 100871 China; 5 Key Laboratory of Reproductive Health, National Health Commission of the People’s Republic of China, Beijing, 100083 China; 6 Department of Epidemiology and Public Health, University College London, London, WC1E 7HB UK; 7 Beijing Famous Cultural Artist Studio--Health Beijing Governance Theory Creation Studio, Beijing, 100872 China

**Keywords:** Migrants, Social integration, Utilization of primary health care

## Abstract

**Background:**

Migrants is a large population in China. To improve the health and wellbeing of migrants is a critical policy and social issue in China, and to enhance the utilization of primary health care by migrants is one of the most important approaches in promoting equity in health. However, there exists little research about the association between social integration and the utilization of primary health care. To address the research gap, this research aims at exploring the relation between social integration and the utilization of primary health care among migrants in China.

**Methods:**

Using the national data from China Migrants Dynamic Survey (CMDS) in 2017, 169,989 migrants were included in this study. Social integration was measured by social communication, acculturation and self-identity, with 8 indicators. The utilization of primary health care was measured by the receiving of health education on infectious diseases (ID) and noncommunicable diseases (NCD) as well as the first visit institution when migrants were sick. After the descriptive statistical analysis, binary logistic regression was employed to evaluate the association between social integration and the utilization of primary health care.

**Results:**

65.99% of the migrants received health education on infectious diseases (ID), 40.11% of the migrants received health education on noncommunicable diseases (NCD) and 8.48% of the migrants chose to go to Community Health Center (CHC) seeking for health services. There was a positive effect of social organization participation, the influence of hometown customs, differences of hygiene habits between migrants and local people, integration willingness and evaluation of identity on the receiving of health education on ID and NCD, as well as a positive effect of civil activities engagement and differences of hygiene habits between migrants and local people on the utilization of CHC after getting sick.

**Conclusions:**

Social integration was associated with the utilization of primary health care among migrants in China. Generally speaking, greater social integration was associated with higher possibility of receiving health education on ID and NCD. However, the effect of social integration on the utilization of CHC was more complex among different indicators. There should be more policy interventions to improve the social integration of migrant which help them to get familiar with the health resource available, as well as improve the capacity of CHC.

## Background

Since the Reform and Opening-up, especially since China established the socialist market economic system, with the process of industrialization and urbanization, the number of migrants in China has increased sharply. The concept *migrates* here refers to China’s floating population rather than international immigrants. International immigrants migrates across national borders, while China’s floating population are those who migrates within the county temporarily for better opportunities for jobs or education. Since the public services that the residents get varies due to the household registration (Hukou) status, the migrants may find it difficult to get sufficient and qualified public services once they migrated away from their household registration place, especially from rural areas to urban cities. According to the Communiqué of the Seventh National Population Census, as of November 1, 2020, China’s migrants has reached 375.8 million, accounting for 26.03% of China’s total population. Compared with 2010, the number of migrants in China increased by 154.4 million, with a growth rate of 69.73%. The migrants have not only promoted China’s social and economic development, but also brings new challenges of the supply of public services. Promoting the equity in utilization of public services and ensure the welfare of the migrants has become an important policy aim of the China’s government.

In the field of health services, Chinese government has issued a large number of policies and measures to promote the accessibility of health services utilized by migrants. One of the most important reforms is to promote universal health coverage through the supply of primary health care. Chinese government has launched basic public health services(BPHS), which are provided free of charge by Community Health Centers(CHC) to all residents in the community, including local residents and migrants. All expenses of the services are paid by public finance. At the same time, the government has increased the investment in CHC to improve their equipment level and service capacity. If the residents seek health services in CHC, the reimbursement proportion will be higher than that in hospitals. Through these measures, the government hopes to guide the residents to seek services in CHC, so as to enhance the equity, accessibility and efficiency of health services supply and reduce the inequity of access to health services between migrants and local residents.

Many studies have focused on the utilization of health services among the migrants. Some studies have shown that compared with local residents, the migrants made less use of health services. For example, Saunders et al. (2021) found that newly arrived migrants utilized less health care than the UK natives, including primary care, outpatient and inpatient care. And the differences in the utilization of the two population was hard to be explained by self-reported health and age [1]. Ginsburg et al(2021) also found that in South Africa, the utilization of health services and chronic medication were different among migrants and non-migrants [2]. Two studies on the utilization of health services among China’s migrants also supported this conclusion [3, 4]. The associated factors of health service utilization of migrants were also one of the focuses of current researches. Current researches generally focus on sociodemographic characteristics, including age, sex, marital status, educational attainment; economic status, including financial source, income, homeownership, etc.; migrating related characteristics, including migrating range, migrating duration, etc. [3–9].In addition, the impact of social relations, social networks and social integration of migrants on health status and health service utilization has gradually attracted the attention of researchers. Although the measurements of social integration varied in different studies, the results of different studies always showed that migrants with higher level of social integration were more likely to adhere to health management behaviors, have less medical return, better health outcomes and better health conditions [10–19].

Since BPHS is an important part of primary health care in China, some scholars have further studied the relationship between social integration and BPHS utilization among migrants. Some scholars carried out researches of specific population. For female migrants in in Changsha, China, social support was associated with the utilization of basic public health services, measured by health record establishment, basic contraceptive service use and cancer screening [20]. Elderly migrants who had local medical insurance, long term settlement intention and over 3 local friends had higher possibility to establish health records in China [21]. In another research of elderly migrants in China, Lin et al(2021) found that among Chinese internal elderly migrants, social contacts mediated the relation between migration characteristics(measured by years of residence and reasons for migration) and the utilization of basic public health services(measured by participation in free health checkups) [22]. Based on national survey, some researches investigated the effect of social integration on utilization of BPHS among domestic migrants in China and found that social integration was related to a higher likelihood of utilization of health records, health education, shorter length of stay in psychiatric hospitals, and less repeated admissions [23–25].

Current researches have shown that for migrants, there is a correlation between social integration and health service utilization. However, the impact of social integration on the utilization of primary health care of migrants is not clear. Considering that health education is an important item of BPHS and Chinese government has always encouraged residents to go to primary health care for health care, in this paper, we use the receiving of health education provided by community healthcare centers and migrants’ first visit institution when they are sick to measure the utilization of basic health services by migrants. This research used national data from China Migrants Dynamic Survey(CMDS) in 2017 to investigate the effect of social integration on the utilization of primary health care to address the knowledge gap.

## Methods

### Study design and data source

China Migrants Dynamic Survey(CMDS) is a nationwide cross-sectional study in China. Data was collected in 2017 in China. The survey was conducted by China’s Health Commission, China population and development research center, Chinese center for disease control and prevention, Health Commission of 31 provinces and Xinjiang Production and Construction Corps in mainland China. The national survey aimed at understanding the living condition and public services utilization of migrants, enhancing the efficiency of related policies. CMDS used a probability proportional to size (PPS)sampling method which is a stratified, multi-stage and proportional scale sampling. The survey covered 436 cities and counties in mainland China. The participants of the survey were migrants aged above 15 years whose household registration is not at the current residence, and had resided in the place for more than a month for working or living. All the participants received an informed consent.

### Data collection

The questionnaire of the survey is designed uniformly, including sociodemographic information, occupation, willingness to migrate or resident and public services utilization. All provinces carried out face-to-face survey with smart phones or pads installed with a specially developed interview system. All the participants are directly interviewed by investigators with unified training. The sample contained 169,989 participants.

### Variables

#### Dependent variables

The dependent variables of this study is the utilization of primary health care among migrants, which has three indicators, the receiving of health education on infectious diseases (ID) and noncommunicable diseases (NCD) as well as the first visit institution when migrants were sick. The receiving of health education was measured by a series of questions including” Have you received health education on prevention of Occupational Diseases/Sexually transmitted diseases and AIDS/pulmonary tuberculosis/chronic disease, reproductive health, smoking control, mental health, Eugenics and Self rescue in public health emergencies?” The answers included “Yes” and “No”. If the participant received at least one kind of health education of the prevention of Sexually transmitted diseases, AIDS or pulmonary tuberculosis, the participant is considered as receiving the health education on the prevention of infectious diseases (ID). If the participant received at least one kind of health education of other 5 kinds, then the participant is considered as receiving the health education on the prevention of noncommunicable diseases (NCD). The receiving of health education on the prevention of infectious diseases (ID) and noncommunicable diseases (NCD) were recoded into two dichotomous variables (1 = “Yes” vs. 0 = “No”).

The first visit institution when migrants are sick is measured by the question “When you were last sick (injured) or unwell, where did you first go to see the illness/injury?” The answers included “community healthcare center (CHC)” and “others”. It was recoded into a dichotomous variable (1 = “community healthcare center” vs. 0 = “others”).

### Independent variable

The independent variable of the study is social integration. Based on the previous researches and the questionnaire of the survey, the social integration of the study encompassed three dimensions, which are social communication, acculturation and self-identity, which is a more comprehensive measure of social integration. Social communication focus on the external environment support for the migrants, especially the social participation, interaction with others and support from organizations. Acculturation focuses more on the attitudes and evaluations of the migrants of the destination, while self-identity mainly measures immigrants’ evaluation of their own identity.

Of 8 indicators of the independent variable, 6 are treated as ordinal variables including civil activities, local medical insurance, preference of residence, the influence of hometown customs on migrants, differences of health habits between migrants and local people, integration willingness and evaluation of identity. Social organizational participation and local medical insurance are treated as nominal variables because the questions in the questionnaire used to measure the social organizational participation are answered in “yes” or “no”, and the question used to measure the local medical insurance is answered in “yes”, “no” or “not clear”, so these two indicators are treated as nominal variables.

**Social communication** includes social organization participation, civil activities engagement and local medical insurance. (1) To measure the social organizational participation, participants were asked if they were a member of any Labor Union, Volunteer Association, Alumni Association, Hometown Chamber of Commerce and any other form of hometown organization. If the participant participated any one kind of organizations above, then the participant is considered. Social organizational participation is dichotomized, with “1” representing the participant participated any kind of organizations above and “0” representing the participant didn’t participate any kind of organizations. (2) Civil activities engagement has four categories with “0” representing the participant has never participated in community activities, activities to advise the government, volunteer activity, or the Party and the League activities, “1” representing the participant occasionally participated any kind of activities, “2” often and “3” always. (3) local medical insurance is measured by asking the participants if they had any kind of medical insurance (0=“No”,1=“Yes” = 1 and 2=“Not clear”).

**Acculturation** consisted of three indicators, including preference of residence, the influence of hometown customs on migrants and differences of health habits between migrants and local people, which were measured by three questions “Do you agree with the statement ‘I like the city/place I live in now’ ” “Do you agree with the statement ‘It is more important for me to do things according to the customs of my hometown’” “Do you agree with the statement ‘My hygiene habits are quite different from those of local citizens’” respectively. Response options included “strongly disagree” = 0, “disagree”=1, “agree”=2 and “strongly agree”=3.

**Self-identity** is composed of two indicators, which were integration willingness and evaluation of identity. The two indicators were measured by the questions “Do you agree with the statement ‘I am willing to integrate into the local people and become one of them’ ” “Do you agree with the statement ‘I think I’m already a local’”. Response options included “strongly disagree” = 0, “disagree”=1, “agree”=2 and “strongly agree”=3.

### Covariates

The utilization of health services of the migrants, along with the effect of social integration on the health and health-seeking behaviors has drawn attention of the researchers recently. In the relevant researches, it has been proved that sociodemographic characteristics including age, sex, education status, marital status were associated with the utilization of health services. In the researches of China’s context, the household registration status(hukou) was also thought to be an important factor that influenced the utilization of health services[26–33]. Addition to the sociodemographic characteristics, Migrant status may also be an important factor because it may affect the migrants’ familiarity with the distribution of local health resources available[34–37]. Referring to existing researches and questionnaire of the survey, we included six sociodemographic characteristics and three migrating related characteristics as covariates as follows.

**Sociodemographic characteristics** We included six sociodemographic characteristics: sex (female or male), age (65 years and above, 55–64 years, 45–54 years, 35–44 years, 25–34 years, 25–34 years, or 15–24 years), regions (east, middle, or west), marital status (married/living with partner or never married/ divorced/widowed), educational attainment (above college degree, college, high school and equivalent or middle school and below), and household registration status (urban or rural).

**Migrating related characteristics** We included three migrating related characteristics: length of migration (1 year and above or less than 1 year), reasons of migration (working/business or other), and range of migration (out of province or in province).

### Statistical analysis

Statistical analyses were carried out by using Stata version 16.0 (StataCorp LLC. Texas, USA). Sociodemographic information, social integration characteristics, and the receiving of health education and the first visit institution of migrants were showed using descriptive statistical analysis, including frequency and proportion. The association between social integration and the receiving of health education as well as the first visit institution were estimated by performing binary logistic regression and using the odds ratio (OR) and 95% confidence intervals (CIs).

## Results

### The sociodemographic and migrating related characteristics

Table 1 shows the sociodemographic and migrating related characteristics distribution of all the migrants who participated the survey. Of all the 169,989 migrants, 51.69%(87,871/169,989) of them were male. 37.81%(64,271/169,989) of the participants were at the age of 25–34, followed by the age group of 35–44(27.31%,46,420/169,989) and 45–54(17.44%,29,652/169,989). Majority of the sampled migrants (82.07%,139,517/169,989) were married or living with partner. 60.7%(103,186/169,989) had an education level of middle school and below. 82.76%(140,687/169,989) of the participants’ household registration status was rural. Majority of the participants migrated to the current residence for the reason of working or business. 77.9%(132,423/169,989) of the migrants has been migrating for more than 1 year. 50.71%(86,199/169,989) of the participants migrated in province. 51.18%(86,995/169,989) of the participants were from the east part of China, 17.06%(28,999/169,989) were from the middle, and 31.76%(53,995/169,989) were from the west.

**Table 1:** The demographic and migrating related characteristics of the sampling migrants in China

	N(%)	Receiving of health education						First Visit Institution(%)	
		Infectious diseases(%)			Noncommunicable diseases(%)				
		No	Yes		No	Yes		CHC	Else
**Sex**									
Male	82,118(48.31)	30,794(35.04)	57,077(64.96)		52,454(59.69)	35,417(40.31)		7379(8.40)	80,492(91.60)
Female	87,871(51.69)	27,023(32.91)	55,095(67.09)		49,347(60.09)	32,771(39.91)		7028(8.56)	75,090(91.44)
**Age**									
15–24	19,038(11.2)	8238(43.27)	10,800(56.73)		12,130(63.71)	6908(36.29)		1797(9.44)	17,241(90.56)
25–34	64,271(37.81)	20,157(31.36)	44,114(68.64)		37,808(58.83)	26,463(41.17)		5976(9.30)	58,295(90.70)
35–44	46,420(27.31)	13,984(30.12)	32,436(69.88)		26,607(57.32)	19,813(42.68)		3808(8.20)	42,612(91.80)
45–54	29,652(17.44)	10,823(36.50)	18,829(63.50)		18,074(60.95)	11,578(39.05)		2034(6.86)	27,618(93.14)
55–64	7390(4.35)	3172(42.92)	4218(57.08)		4945(66.91)	2445(33.09)		536(7.25)	6854(92.75)
65-	3218(1.89)	1443(44.84)	1775(55.16)		2237(69.52)	981(30.48)		256(7.96)	2962(92.04)
**Marital status**									
Never married/ Divorced/widowed	30,472(17.93)	13,069(42.89)	17,403(57.11)		18,871(61.93)	11,601(38.07)		2604(8.55)	27,868(91.45)
Married/living with partner	139,517(82.07)	44,748(32.07)	94,769(67.93)		82,930(59.44)	56,587(40.56)		11,803(8.46)	127,714(91.54)
**Educational attainment**									
<=Middle school	103,186(60.7)	37,198(36.05)	65,988(63.95)		63,797(61.83)	39,389(38.17)		7834(7.59)	95,352(92.41)
High school or equivalent	37,224(21.9)	11,657(31.32)	25,567(68.68)		21,027(56.49)	16,197(43.51)		3282(8.82)	33,942(91.18)
College	28,687(16.88)	8659(30.18)	20,028(69.82)		16,381(57.10)	12,306(42.90)		3180(11.09)	25,507(88.91)
>College	892(0.52)	303(33.97)	589(66.03)		596(66.82)	296(33.18)		111(12.44)	781(87.56)
**Household registration status**									
Agricultural	140,687(82.76)	48,518(34.49)	92,169(65.51)		84,778(60.26)	55,909(39.74)		11,495(8.17)	129,192(91.83)
Nonagricultural	29,302(17.24)	9299(31.74)	20,003(68.26)		17,023(58.10)	12,279(41.90)		2912(9.94)	26,390(90.06)
**Reasons of migration**									
Other	27,872(16.4)	9327(33.46)	18,545(66.54)		17,016(61.05)	10,856(38.95)		2370(8.50)	25,502(91.50)
Working or business	142,117(83.6)	48,490(34.12)	93,627(65.88)		84,785(59.66)	57,332(40.34)		12,037(8.47)	130,080(91.53)
**Duration**									
<=1 year	37,566(22.1)	21,585(57.46)	15,981(42.54)		27,715(73.78)	9851(26.22)		3315(8.82)	34,251(91.18)
> 1 year	132,423(77.9)	36,232(27.36)	96,191(72.64)		74,086(55.95)	58,337(44.05)		11,092(8.38)	121,331(91.62)
**Range of migration**									
In province	86,199(50.71)	25,580(29.68)	60,619(70.32)		47,963(55.64)	38,236(44.36)		7001(8.12)	79,198(91.88)
Out of province	83,790(49.29)	32,237(38.47)	51,553(61.53)		53,838(64.25)	29,952(35.75)		7406(8.84)	76,384(91.16)
**Region**									
East	86,995(51.18)	33,658(38.69)	53,337(61.31)		59,622(68.53)	27,373(31.47)		9511(10.93)	77,484(89.07)
Middle	28,999(17.06)	8435(29.09)	20,564(70.91)		16,532(57.01)	12,467(42.99)		2135(7.36)	26,864(92.64)
West	53,995(31.76)	15,724(29.12)	38,271(70.88)		25,647(47.50)	28,348(52.50)		2761(5.11)	51,234(94.89)

Notes: CHC, Community Health Center

### Social integration

As Table 2 shows, of all participants, 44.32%(75,341/169,989) were a member of the Labor Union, Volunteer Association, Alumni Association, Hometown Chamber of Commerce or any other form of organizations, 55.68%(94,648/169,989) never joined any organizations mentioned above. As far as the civil activities engagement, 95.27%(161,951/169,989) of the participants often participated in community activities, activities to advise the government, volunteer activity, or the Party and the League activities, 4.27%(7261/169,989) had never participates any activities above, and 0.45%(777/169,989) had never or just occasionally participated any kind of activities.

**Table 2:** Receiving of health education and first visit institution across different type of social integration status among the sampling migrants in China

	N(%)	Receiving of health education						First Visit Institution (%)	
		Infectious diseases (%)			Noncommunicable diseases (%)				
		No	Yes		No	Yes		CHC	Else
**Social communication**									
Social organization participation									
No	94,648(55.68)	40,646(42.94)	54,002(57.06)		64,209(67.84)	30,439(32.16)		7958(8.41)	86,690(91.59)
Yes	75,341(44.32)	17,171(22.79)	58,170(77.21)		37,592(49.90)	37,749(50.10)		6449(8.56)	68,892(91.44)
Civil activities engagement									
No	415(0.24)	59(14.22)	356(85.78)		110(26.51)	305(73.49)		13(3.13)	402(96.87)
Occasionally	362(0.21)	71(19.61)	291(80.39)		115(31.77)	247(68.23)		7(1.93)	355(98.07)
Sometimes	161,951(95.27)	56,152(34.67)	105,799(65.33)		98,220(60.65)	63,731(39.35)		13,678(8.45)	148,273(91.55)
Usually	7261(4.27)	1535(21.14)	5726(78.86)		3356(46.22)	3905(53.78)		709(9.76)	6552(90.24)
Local medical insurance									
No	24,656(14.5)	7119(28.87)	17,537(71.13)		14,366(58.27)	10,290(41.73)		2803(11.37)	21,853(88.63)
Yes	3457(2.03)	1426(41.25)	2031(58.75)		2297(66.44)	1160(33.56)		387(11.19)	3070(88.81)
Not clear	141,876(83.46)	49,272(34.73)	92,604(65.27)		85,138(60.01)	56,738(39.99)		11,217(7.91)	130,659(92.09)
**Acculturation**									
Preference of residence									
Strongly disagreed	75,528(44.43)	23,169(30.68)	52,359(69.32)		42,463(56.22)	33,065(43.78)		6620(8.76)	68,908(91.24)
Disagreed	92,019(54.13)	33,873(36.81)	58,146(63.19)		57,953(62.98)	34,066(37.02)		7590(8.25)	84,429(91.75)
Agreed	1485(0.87)	556(37.44)	929(62.56)		938(63.16)	547(36.84)		115(7.74)	1370(92.26)
Strongly agreed	957(0.56)	219(22.88)	738(77.12)		447(46.71)	510(53.29)		82(8.57)	875(91.43)
Influence of hometown customs									
Strongly disagreed	59,231(34.84)	19,454(32.84)	39,777(67.16)		34,676(58.54)	24,555(41.46)		5377(9.08)	53,854(90.92)
Disagreed	71,977(42.34)	25,329(35.19)	46,648(64.81)		44,233(61.45)	27,744(38.55)		5722(7.95)	66,255(92.05)
Agreed	16,659(9.8)	5005(30.04)	11,654(69.96)		8943(53.68)	7716(46.32)		1429(8.58)	15,230(91.42)
Strongly agreed	22,122(13.01)	8029(36.29)	14,093(63.71)		13,949(63.05)	8173(36.95)		1879(8.49)	20,243(91.51)
Differences of health habits between migrants and local people									
Strongly disagreed	91,648(53.91)	31,319(34.17)	60,329(65.83)		55,410(60.46)	36,238(39.54)		7894(8.61)	83,754(91.39)
Disagreed	28,101(16.53)	11,206(39.88)	16,895(60.12)		18,004(64.07)	10,097(35.93)		1917(6.82)	26,184(93.18)
Agreed	44,537(26.2)	13,160(29.55)	31,377(70.45)		24,870(55.84)	19,667(44.16)		4208(9.45)	40,329(90.55)
Strongly agreed	5703(3.35)	2132(37.38)	3571(62.62)		3517(61.67)	2186(38.33)		388(6.80)	5315(93.20)
**Self-identity**									
Integration willingness									
Strongly disagreed	9945(5.85)	4585(46.10)	5360(53.90)		7122(71.61)	2823(28.39)		965(9.70)	8980(90.30)
Disagreed	88,307(51.95)	32,031(36.27)	56,276(63.73)		54,990(62.27)	33,317(37.73)		7090(8.03)	81,217(91.97)
Agreed	1719(1.01)	753(43.80)	966(56.20)		1131(65.79)	588(34.21)		160(9.31)	1559(90.69)
Strongly agreed	70,018(41.19)	20,448(29.20)	49,570(70.80)		38,558(55.07)	31,460(44.93)		6192(8.84)	63,826(91.16)
Evaluation of his/her identity									
Strongly disagreed	35,405(20.83)	14,972(42.29)	20,433(57.71)		24,686(69.72)	10,719(30.28)		3780(10.68)	31,625(89.32)
Disagreed	86,635(50.97)	28,713(33.14)	57,922(66.86)		51,216(59.12)	35,419(40.88)		6867(7.93)	79,768(92.07)
Agreed	5304(3.12)	2251(42.44)	3053(57.56)		3662(69.04)	1642(30.96)		579(10.92)	4725(89.08)
Strongly agreed	42,645(25.09)	11,881(27.86)	30,764(72.14)		22,237(52.14)	20,408(47.86)		3181(7.46)	39,464(92.54)

As far as local medical insurance, 2.03%(3457/169,989) of the participants had local medical insurance, 14.50%(24,656/169,989) didn’t have any kind of local medical insurance, and 83.46%(141,876/169,989) were not clear of local medical insurance attainment.

As far as the preference for place of residence, 44.43%(75,528/169,989) of the participants strongly disagree with the statement “I like the city/place I live in now”, 54.13%(92,019/169,989) disagree with the statement. Only 1.43%(2442/169,989) of the participants agree or strongly agree with the statement. Considering the influence of hometown customs on migrants, 34.84%(59,231/169,989) strongly disagree with the statement “It is more important for me to do things according to the customs of my hometown”0.42.34%(71,977/169,989) disagree with the statement. 9.80%(16,659/169,989) of the participants agree and 13.01%(22,122/169,989) strongly agree with the statement. In terms of the differences of health habits between migrants and local people, 53.91%(91,648/169,989) strongly disagree with the statement “My hygiene habits are quite different from those of local citizens”0.16.53%(28101/169989) disagree with the statement. 26.20%(44537/169989) of the participants agree and 3.35%(5703/169989) strongly agree with the statement.

Considering the integration willingness, 5.85%(9945/169,989) strongly disagree with the statement “I am willing to integrate into the local people and become one of them”, 51.95%(88,307/169,989) disagree with the statement. 1.01%(1719/169,989) agree and 41.19%(70,018/169,989) strongly agree with the statement. As far as the evaluation of identity, 20.83%(35,405/169,989) of the participants strongly disagree with the statement “I think I’m already a local”, 50.97%(86,635/169,989) disagree with the statement, 3.12%(5304/169,989) agree and 25.09%(42,645/169,989) strongly agree with the it.

### Association between social integration and utilization of primary health care

Table 3 shows the regression results of association between social integration and utilization of primary health care after controlling the confounding variables. Receiving of health education on the prevention of infectious diseases(ID) is associated with social organizational participation(aOR = 2.5; 95% CI,2.50–2.61). Compared with migrants who never participated any civil activities, the odds of receiving the health education on the prevention of ID of migrants who participated were 0.59(95% CI, 0.40–0.89), 0.36(95% CI, 0.27–0.48), 0.47(95% CI, 0.35–0.63). Compared with migrants didn’t have local medical insurance, migrants who had or not clear of the attainment of medical insurance had lower possibility to receive health education of ID(aOR = 0.76; 95% CI, 0.70–0.82; aOR = 0.92;95% CI, 0.89–0.96, respectively). Compared with migrants who strongly dislike the city/place they live in now, those who disagreed had higher possibility to receive the health education of ID(aOR = 0.95; 95% CI,0.92–0.97). Besides, the smaller the influence of hometown custom on migrants, the more likely the migrants receive the health education on the prevention of ID(aOR = 1.13; 95% CI,1.08–1.19; aOR = 1.14; 95% CI,1.10–1.18; aOR = 1.16, 95% CI,1.12–1.20). Compared with migrants who strongly agreed that their hygiene habits were quite different from those of local citizens, migrants who agreed or strongly agreed had higher possibility to receive health education on the prevention of ID(aOR = 1.25; 95% CI,1.17–1.33; aOR = 1.14; 95% CI,1.07–1.21, respectively). Migrants with higher willingness to integrate into the local people and become one of them were more likely to receive the health education of ID(aOR = 1.17; 95% CI,1.12–1.23; aOR = 1.27; 95% CI,1.21–1.34, respectively). Compared with migrants who strongly disagreed that they were a local, those who with higher local identification had higher possibility to receive the health education of ID(aOR = 1.22; 95% CI,1.18–1.25; aOR = 1.25; 95% CI,1.21–1.30, respectively).

**Table 3:** Associations between social integration and utilization of primary health care among migrates in China

	Receiving of health education								First Visit Institution			
	Infectious diseases				Noncommunicable diseases							
	Univariable analysis		Multivariable analysis		Univariable analysis		Multivariable analysis		Univariable analysis		Multivariable analysis	
	cOR(95% CI)	*P*	aOR(95% CI)	*P*	cOR(95% CI)	*P*	aOR(95% CI)	*P*	cOR(95% CI)	*P*	aOR(95% CI)	*P*
**Social communication**												
Social organization participation												
No	1(Ref)		1(Ref)		1(Ref)		1(Ref)		1(Ref)		1(Ref)	
Yes	2.55(2.50–2.61)	< 0.001	2.50(2.45–2.56)	< 0.001	2.12(2.08–2.16)	< 0.001	2.02(1.98–2.06)	< 0.001	1.02(0.99–1.06)		0.94(0.91–0.98)	< 0.001
Civil activities engagement												
No	1(Ref)		1(Ref)		1(Ref)		1(Ref)		1(Ref)		1(Ref)	
Occasionally	0.68(0.47–0.99)	< 0.01	0.59(0.40–0.89)	< 0.005	0.78(0.57–1.06)		0.68(0.49–0.94)	< 0.01	0.61(0.24–1.55)		0.60(0.24–1.53)	
Sometimes	0.31(0.24–0.41)	< 0.001	0.36(0.27–0.48)	< 0.001	0.23(0.19–0.29)	< 0.001	0.28(0.22–0.35)	< 0.001	2.85(1.64–4.96)	< 0.001	2.36(1.35–4.11)	< 0.001
Usually	0.62(0.47–0.82)	< 0.001	0.47(0.35–0.63)	< 0.001	0.42(0.34–0.52)	< 0.001	0.36(0.28–0.45)	< 0.001	3.35(1.92–5.85)	< 0.001	2.61(1.49–4.58)	< 0.001
Local medical insurance												
No	1(Ref)		1(Ref)		1(Ref)		1(Ref)		1(Ref)		1(Ref)	
Yes	0.58(0.54–0.62)	< 0.001	0.76(0.70–0.82)	< 0.001	0.71(0.65–0.76)	< 0.001	0.80(0.74–0.87)	< 0.001	0.98(0.88–1.10)		1.06(0.94–1.19)	
Not clear	0.76(0.74–0.79)	< 0.001	0.92(0.89–0.96)	< 0.001	0.93(0.91–0.96)	< 0.001	1.01(0.98–1.05)		0.67(0.64–0.70)	< 0.001	0.86(0.82–0.90)	< 0.001
**Acculturation**												
Preference of residence												
Strongly agreed	1(Ref)		1(Ref)		1(Ref)		1(Ref)		1(Ref)		1(Ref)	
Disagreed	0.76(0.74–0.78)	< 0.001	0.95(0.92–0.97)	< 0.001	0.76(0.74–0.77)	< 0.001	0.91(0.89–0.94)	< 0.001	0.94(0.90–0.97)	< 0.001	0.96(0.91-1.00)	< 0.05
Agreed	0.74(0.67–0.82)	< 0.001	1.05(0.93–1.19)		0.75(0.67–0.83)	< 0.001	0.91(0.80–1.03)		0.87(0.72–1.06)		0.85(0.69–1.04)	
Strongly agreed	1.49(1.28–1.74)	< 0.001	1.06(0.89–1.26)		1.47(1.29–1.67)	< 0.001	1.06(0.92–1.22)		0.98(0.78–1.23)		0.91(0.71–1.16)	
Influence of hometown customs												
Strongly agreed	1(Ref)		1(Ref)		1(Ref)		1(Ref)		1(Ref)		1(Ref)	
Agreed	1.33(1.27–1.39)	< 0.001	1.13(1.08–1.19)	< 0.001	1.47(1.41–1.53)	< 0.001	1.30(1.25–1.36)	< 0.001	1.01(0.94–1.09)		0.91(0.84–0.98)	< 0.01
Disagreed	1.05(1.02–1.08)	< 0.001	1.14(1.10–1.18)	< 0.001	1.07(1.04–1.10)	< 0.001	1.17(1.13–1.21)	< 0.001	0.93(0.88–0.98)	< 0.001	0.93(0.88–0.99)	< 0.01
Strongly disagreed	1.17(1.13–1.20)	< 0.001	1.16(1.12–1.20)	< 0.001	1.21(1.17–1.25)	< 0.001	1.24(1.20–1.29)	< 0.001	1.08(1.02–1.14)	< 0.001	1.01(0.95–1.07)	
Differences of health habits between migrants and local people												
Strongly agreed	1(Ref)		1(Ref)		1(Ref)		1(Ref)		1(Ref)		1(Ref)	
Agreed	1.42(1.34–1.51)	< 0.001	1.25(1.17–1.33)	< 0.001	1.27(1.20–1.35)	< 0.001	1.18(1.11–1.26)	< 0.001	1.43(1.28–1.59)	< 0.001	1.29(1.15–1.44)	< 0.001
Disagreed	0.90(0.85–0.96)	< 0.001	0.99(0.93–1.06)		0.90(0.85–0.96)	< 0.001	1.03(0.97–1.10)		1.00(0.90–1.12)		0.99(0.88–1.12)	
Strongly disagreed	1.15(1.09–1.22)	< 0.001	1.14(1.07–1.21)	< 0.001	1.05(1.00-1.11)	< 0.05	1.13(1.06–1.20)	< 0.001	1.29(1.16–1.44)	< 0.001	1.14(1.02–1.27)	< 0.01
**Self-identity**												
Integration willingness												
Strongly disagreed	1(Ref)		1(Ref)		1(Ref)		1(Ref)		1(Ref)		1(Ref)	
Disagreed	1.50(1.44–1.57)	< 0.001	1.17(1.12–1.23)	< 0.001	1.53(1.46–1.60)	< 0.001	1.21(1.15–1.27)	< 0.001	0.81(0.76–0.87)	< 0.001	0.89(0.83–0.96)	< 0.001
Agreed	1.10(0.99–1.22)	< 0.05	0.90(0.80–1.02)		1.31(1.18–1.46)	< 0.001	1.14(1.01–1.28)	< 0.01	0.96(0.80–1.14)		1.03(0.85–1.24)	
Strongly agreed	2.07(1.99–2.16)	< 0.001	1.27(1.21–1.34)	< 0.001	2.06(1.97–2.16)	< 0.001	1.28(1.22–1.35)	< 0.001	0.90(0.84–0.97)	< 0.001	1.00(0.93–1.09)	
Evaluation of identity												
Strongly disagreed	1(Ref)		1(Ref)		1(Ref)		1(Ref)		1(Ref)		1(Ref)	
Disagreed	1.48(1.44–1.52)	< 0.001	1.22(1.18–1.25)	< 0.001	1.59(1.55–1.64)	< 0.001	1.32(1.28–1.36)	< 0.001	0.72(0.69–0.75)	< 0.001	0.77(0.74–0.81)	< 0.001
Agreed	0.99(0.94–1.05)		1.01(0.95–1.08)		1.03(0.97–1.10)		1.02(0.95–1.09)		1.03(0.93–1.13)		1.02(0.93–1.12)	
Strongly agreed	1.90(1.84–1.96)	< 0.001	1.25(1.21–1.30)	< 0.001	2.11(2.05–2.18)	< 0.001	1.45(1.40–1.51)	< 0.001	0.67(0.64–0.71)	< 0.001	0.66(0.62–0.70)	< 0.001

Notes: cOR, crude odds ratio; aOR, adjusted odds ratio

As far as the receiving of health education on the prevention of noncommunicable diseases (NCD), compared with migrants who didn’t participate in social organizations, migrants that participated in social organizations were more likely to receive health education on NCD(aOR = 2.02; 95% CI,1.98–2.06). Compared with migrants whose hometown customs has strong influence on them, other migrants who believed that there was less influence of hometown customs had higher possibility to receive health education(aOR = 1.30; 95% CI,1.25–1.36; aOR = 1.17; 95% CI,1.13–1.21; aOR = 1.24, 95% CI,1.20–1.29). Meanwhile, migrants who thought the differences of health habits between them and local people are smaller tended to receive health education on NCD(aOR = 1.18; 95% CI,1.11–1.26; aOR = 1.03; 95% CI,0.97–1.10; aOR = 1.13, 95% CI,1.06–1.20). Compared with migrants who were strongly unwilling to integrate into the local people and become one of them, migrants with higher willingness were more likely to receive health education on NCD(aOR = 1.21; 95% CI,1.15–1.27; aOR = 1.14; 95% CI,1.01–1.28; aOR = 1.28, 95% CI,1.22–1.35). Compared with migrants that strongly didn’t think he/she was already a local, migrants who identified he/she a local had higher possibility to receive health education on NCD(aOR = 1.32; 95% CI,1.28–1.36; aOR = 1.02; 95% CI,0.95–1.09; aOR = 1.45, 95% CI,1.40–1.51).

As far as the first visit institution when migrants were sick, compared with migrants participated in social organizations, those who didn’t were less likely to go to CHC(aOR = 0.94; 95% CI,0.91–0.98). In relation to migrants without health insurance, migrants who were not aware of the health insurance attainment were more likely to go to CHC(aOR = 0.86; 95% CI,0.82–0.90). Migrants who had higher preference for place of residence tended not go to CHC(aOR = 0.96; 95% CI,0.91-1.00). Migrants on whom the influence of hometown customs were smaller had smaller possibility to go to CHC(aOR = 0.91; 95% CI,0.84-0.98-4.11; aOR = 0.93; 95% CI,0.88–0.99).Migrants with higher integration willingness were less likely to go to CHC(aOR = 0.89; 95% CI,0.83–0.96). Migrants who more thought of himself or herself as local were less likely to go to CHC(aOR = 0.77; 95% CI,0.74–0.81; aOR = 0.66; 95% CI,0.62–0.70). Compared with migrants didn’t engaging in civil activities, migrants engaging in civil activities were more likely to go to CHC(aOR = 2.36; 95% CI,1.35–4.11; aOR = 2.61; 95% CI,1.49–4.58). Compared with migrants thought their hygiene habits were quite different from local citizens, migrants who thought the differences were smaller tended to go to CHC(aOR = 1.29; 95% CI,1.15–1.44; aOR = 1.14; 95% CI,1.02–1.27).

## Discussion

Migrants accounts for 26.03% of China’s total population, and the health of migrants is an important policy issue as well as social issue, and many policies has been complimented to improve the accessibility of health services. The equitable access to primary health care is one of the critical measures. Primary health care is believed to be the most cost-effectiveness way to improve the accessibility of health care. So it’s necessary to study the utilization of primary health care of migrants, and the effect of social integration on it should be studied since social integration is an important variable that helps to understand the health services seeking behavior of migrants.

Social integration is a complex concept and can be realized and measured using many dimensions and indicators, and this research focuses on social communication (composed of social organizational participation, civil activities engagement and local medical insurance), acculturation (composed of preference for place of residence, the influence of hometown customs and differences of health habits between migrants and local people) and self-identity (composed of integration willingness and evaluation of identity)to evaluate the association between social integration and utilization of primary health care. And It’s clear that the associations between different indicators of social integration and utilization of primary health care were different, which implied that the inherent effect of social integration on the utilization of primary health care is complicated and can’t be understood in a single path.

As we can see, through our research of the association between social integration and the willingness to receive health education, we found that generally speaking, acculturation and self-identity had positive effect on the receiving of health education, migrants who thought the influence of hometown customs were less and the difference of hygiene habits between them and local people were smaller had higher possibility to receive higher education. This could be the results of migrants adapting to local life and customs. With the improvement of acculturation to local life, migrants were more likely to utilize health services. Meanwhile, as migrants were willing to integrate into local people and become one of them, or already thought themselves as local, they tended receive health education, which may because identity enhanced their willingness to utilize local basic public health services. However, there exists heterogeneity between the association between social communication and the receiving of health education. Migrants who participated in social organizations were more likely to receive health education, and this may because in the process of joining in different social organizations, migrants got access to more knowledge of the importance of keeping healthy, and their awareness of receiving health education has been improved. we found that civil activities had a negative effect on the possibility of health education receiving, which may be a result of lack of time since civil activities were usually time-consuming. Meanwhile, migrates who were willing to take part in civil activities may have better health condition, resulting in the ignoring of health eduction. The attainment of local health insurance also had an negative effect and this may because that health insurance decreased migrants’ health expenditure, causing their lack of attention to health education.

As far As the first visit institution, this research found that many indicators of social integration, including social organizational participation, local medical insurance, preference for place of residence, the influence of hometown customs, integration willingness, evaluation of identity had an negative effect on the health services utilization when migrants were sick. This could result from the situation that the hierarchical medical system hasn’t realized in China, and residents could go to any health institution seeking for health services. With higher level of social integration, migrants may get familiar with health resources, and tended to go to public hospitals for health services instead of CHC. This was because that compared with CHC, public hospitals usually have higher service capacities, advanced medical equipment and well educated medical staff. Also, by 2021, the coverage of China’s Basic Medical Insurance has reached 95%, which reduced the medical expenditure of residents and stimulated them to seek services in public hospitals instead of CHC to some extent.

What’s more, the utilization of primary health care of migrants provided a lens to examine the health reform in China. In fact, to improve the utilization of primary health care provided by CHC has always been an very important policy objective since the new medical reform in 2009. The Healthy China Strategy also call for more effective policies and strategies to achieve health equity. China’s government has issued many policies to improve the capacity of CHC and guide residents to seek health services in CHC instead of going to hospitals directly. The most influential policy is the integrated healthcare reform and the hierarchical health system reform, which aims at enhancing network governance and inter-organizational collaboration between health organizations. Through the integration of health services and the re-arrange of the responsibility of different health organizations, health resources were expected to be re-allocated more to CHC so that the services capacity may be improved, attracting the residents to seek for health services in CHC. Meanwhile, the reimbursement of health expenditure is usually higher than that of hospitals if a patient goes to CHC for healthcare. One of the government targets is that all residents go to CHC for healthcare first and then referred to hospitals by CHC’s physicians if needed. However, according to our research, only 8.48% migrants chose CHC as the first visit institution when they were sick. We think the gap between policy target and the reality have two reasons. First, the capacity of CHC is still poorer than hospitals, and the income of CHC’s physicians is much lower than that of doctors in hospitals. As a result, medical graduates are often unwilling to seek employment at CHC due to the low salary and poor career development, which in turn hampered the improvement of capacity of CHC. Second, although from the new medical reform, the capacity of CHC has been largely improved, since the migrants are always unfamiliar with the health institutions of the destination, they tend to go to the hospitals with better organizational reputation instead of CHC. In this case, to improve social integration of the migrants makes them having better understanding of health resources available and improve the access of primary healthcare.

This article showed that generally, social integration had positive effect on the receiving of health education on ID and NCD, but the effect of social integration on the first visit institution when migrants were sick was more complex. However, we think it’s still very important to improve the social integration of migrants since this may help migrants learn more about the health resource available and reduce the obstacles in the process of obtaining health services. What’s more, the utilization of primary health care is also a very complicated policy issue involving the capacity of health organizations, resource allocation within the health system, and public attitudes towards CHC. So, we should view the association of social integration and utilization of primary health care in a broader vision and all policies and strategies should take the social integration of the migrants into account. From the perspective of the whole health system, more equitable health services should be provided to all residents of the community, no matter the local residents or the migrants. In China, CHC are responsible to provide basic public health services(BPHS). CHC should establish health records for the migrants in time, so that CHC, other health institutions and health administrative departments can timely learn about the health information of the migrants. From the perspective of the individuals, it’s very important to change the health behaviors of the migrants, which can be affected by the attitude and motivation of the individuals. So, it might be helpful if the different departments of the society such as civil affair department and education departments can work together to promote health education and provide detailed policies about primary healthcare, which may help the migrants to get more comprehensive and continuous health care.

However, this research has two potential limitations. First, since the measure of all the variables were collected based on the self-report data, so there may existed recall bias when participants recalled his/her utilization of primary health care and social integration characteristics. Second, some factors, for example, economic status of migrants were not included in the original data set, which may cause a bias in the process of exploring the relation between social integration and utilization of health services. To handle this problem, we used the variable *region* to substitute for the effect of economic status since the economic development of the west, middle and east region of China are different.

## Conclusions

Social integration, which composed of many dimensions and indicators were associated with the utilization of primary health services including the receiving of health education and the first visit institution getting sick after controlling the confounding factors. To improve the integration status of migrants may enhance the utilization of primary health care, but this should be embedded with other measures improving the capacity of CHC.

## Data Availability

The dataset supporting the conclusions of this article was acquired at https://www.ncmi.cn/phda/dataDetails.do?id=CSTR:A0006.11.A000T.201906.000225.

## Abbreviations

ID: Infectious diseases
NCD: Noncommunicable diseases
CHC: Community Health Center
BPHS: Basic Public Health Services
CMDS: China Migrants Dynamic Survey
PPS: probability proportional to size ()
OR: Odds ratio
cOR: Crude odds ratio
aOR: Adjusted odds ratio
CIs: Confidence intervals
